# Cognitive assessment in an elderly population with metabolic syndrome in Brazil

**DOI:** 10.1590/S1980-57642013DN70200011

**Published:** 2013

**Authors:** Nadia Shigaeff, Alessandro Ferrari Jacinto, Fabio Gazelato de Mello Franco, Gabriela Chiochetta, Maysa Seabra Cendoroglo, Vanessa de Albuquerque Cítero

**Affiliations:** 1 Universidade Federal de São Paulo, São Paulo SP, Brazil;; 2 Hospital Israelita Albert Einstein, São Paulo SP, Brazil.

**Keywords:** metabolic syndrome X, neuropsychological tests, cognition disorders, memory, attention, elderly

## Abstract

Chronic degenerative conditions are very common in the elderly. According to medical literature, there is a correlation between cognitive impairment among elders and arterial hypertension/hyperglycemia which in turn are common diseases among the elderly population worldwide. Nonetheless, data on the association between cognitive impairment and Metabolic Syndrome (MetS) remains controversial.

**OBJECTIVE:**

To compare the cognitive status of Brazilian elderly outpatients with and without MetS.

**METHODS:**

A cross-sectional case-control study with 49 subjects (25 MetS and 24 controls) who underwent a global geriatric and neuropsychological assessment was carried out. The scores for cognitive abilities (sustained attention, alternating attention, immediate memory, working memory, memory - immediate recall, memory - delayed recall, memory - recognition, executive function, ideomotor praxis, constructive praxis, naming ability, verbal fluency) were compared with the data for the normal population and differences between case and control groups were analyzed using Student's t-test or the Mann-Whitney test.

**RESULTS:**

Forty-five patients (91.8%) were female, with a mean age of 73.9±5.9 years, and 3.0±1.0 years of schooling. A significant difference (p<0.01) was found between case and control groups regarding the MetS components. For cognitive abilities, no statistically significant difference was detected between the groups and all subjects presented low cognitive scores.

**CONCLUSION:**

The results obtained in the present study showed that MetS was not associated with cognitive impairment in this population. Further prospective studies are necessary to investigate the influence of well-controlled MetS on cognitive performance among elders.

## Abstract (pt)

Quadros crônico-degenerativos são prevalentes na população idosa. Estudos prévios tem correlacionado prejuízo cognitivo em idosos com hipertensão e hiperglicemia que por sua vez são doenças bastante comuns do envelhecimento na população mundial. Apesar disso ainda existem dados controversos sobre a associação entre prejuízo cognitivo e síndrome metabólica (SMet).

**OBJETIVO:**

Comparar o estado cognitivo de pacientes brasileiros idosos em atendimento ambulatorial com e sem SMet.

**MÉTODOS:**

Estudo de caso-controle transversal com 49 pacientes (25 com SMet e 24 controles) que foram submetidos à avaliação geriátrica global e neuropsicológica. Os escores de habilidades cognitivas (atenção sustentada, atenção alternada, memória imediata, memória operacional, memória tardia - evocação imediata, tardia e de reconhecimento -, função executiva, praxia ideomotora, praxia visuo-construtiva, habilidade de nomeação e fluência verbal) foram comparados com dados da população saudável e a diferença entre caso e controle foi analisada usando o teste t de Student ou o teste de Mann-Whitney.

**RESULTADOS:**

Quarenta e cinco pacientes (91.8%) foram mulheres com idade média de 73.9±5.^9^ anos e escolaridade média de 3.0±1.0anos. Observou-se uma diferença significativa (p<0.01) entre os grupos com relação aos critérios da SMet. Com relação às habilidades cognitivas não houve diferença estatística entre os grupos e todos os pacientes apresentaram baixo escore cognitivo.

**CONCLUSÃO:**

Os resultados obtidos no presente estudo indicaram que a SMet não foi associada com o prejuízo cognitivo nesta população. Outros estudos prospectivos são necessários para melhor investigar a influência de idosos bem compensados nos componentes da SMet no desempenho cognitivo ao longo do tempo.

## INTRODUCTION

The Brazilian population has aged in the last few decades.^1,2^ Chronic degenerative diseases, such as dementia, are highly prevalent in the elderly. The identification of risk factors for cognitive impairment allows early detection of dementia states.^3^

Previous studies have shown correlation between cognitive impairment, arterial hypertension and serum hyperglycemia^4,5^ which are common diseases in the elderly population worldwide. Other studies have shown that elderly individuals with Metabolic Syndrome (MetS) have poor cognitive performance compared with subjects without MetS, especially on information processing speed, memory (immediate and delayed recall), mental flexibility, and also exhibit low scores on the Mini Mental State Examination (MMSE).^6-8^ These studies have also concluded that hyperglycemia is the main MetS factor involved in cognitive impairment.

According to the American Heart Association and the National Heart, Lung, and Blood Institute,^9^, the diagnosis of MetS has to meet three of the following five criteria: abdominal obesity defined as waist circumference >102 cm in men and >88 cm in women; serum triglycerides ≥150 mg/dL or specific drug treatment; serum HDL-cholesterol <40 mg/dL in men and <50 mg/dL in women or specific drug treatment; arterial blood pressure ≥130/85 mmHg or specific drug treat- ment and fasting plasma glucose ≥100 mg/dL or specific drug treatment. Although each of the five criteria of MetS negatively influences cognition, it remains unclear whether MetS is a risk factor.^10^

The aim of the present study was to compare the cognitive status of Brazilian elderly outpatients with and without MetS.

## METHODS

Subjects for this study were recruited from an Outpatient Geriatrics Service at the Hospital Israelita Albert Einstein and from the Universidade Federal de São Paulo, both situated in São Paulo, Brazil. All subjects agreed to participate in a one-year case-control follow-up which aimed to characterize the role of MetS in cerebral perfusion and cognition. The present manuscript is part of this research and reports cross-sectional data. The project was approved by the Institutional Research and Ethics Board of the Hospital Israelita Albert Einstein and of the Universidade Federal de São Paulo (process number: 09/1154 and 0306/10, respectively).

The elderly, all of whom were routinely followed at a MetS outpatient service, were invited to participate (N=37). The group comprised low-income individuals attended by the Brazilian Public Health Care System. The following inclusion criteria were adopted: be at least 65 years old, have 1 to 4 years of schooling and a score of 22 or higher on the Mini Mental State Examination (MMSE).^11^ The exclusion criteria were patients diagnosed with other neurodegenerative conditions, cerebrovascular disease or major psychiatric disorders. The final sample consisted of 25 subjects with MetS. There was no statistically significant difference between the included and excluded MetS subjects regarding gender and age.

Twenty-four healthy subjects with matched epidemiological characteristics and criteria were invited to participate in the control group. The control subjects attended community recreation centers for the elderly.

Subjects underwent global geriatric and neuropsychological assessment which included patients' medical background, a clinical assessment, blood tests and the neuropsychological tests described in Chart 1. These tests^12^ evaluate 12 abilities of cognition; the raw scores of the neuropsychological tests were weighted by age and schooling and converted into Z-scores (regarding RAVLT, gender was also considered). The Wechslers Digit Span was converted in weighted score according to the Wechslers Manual which presents the results weighted by age.

**Chart 1:** Description of 12 cognitive abilities evaluated by the eight tests applied in the study.

Abilities	Description	Tests
Sustained Attention	Capacity to maintain an attentional activity over a period of time	Trail Making Test -TMT (Part A)
Alternating Attention	Capacity to shift in focus and tasks	Trail Making Test - TMT (Part B)
Immediate Memory	Capacity to grasp an adequate amount of information and recall it immediately	Digit Span - Direct Order (WAIS-III)
Working Memory	Capacity to hold and manipulate information in mind for a short period of time	Digit Span - Reverse Order (WAIS-III)
Memory - Immediate Recall	Capacity to recall the information immediately after the stimulus	Rey Auditory Verbal Learning - RAVLT
Memory - Delayed Recall	Capacity to recall the information after a short period, usually 20 minutes after the stimulus	Rey Auditory Verbal Learning - RAVLT
Memory - Recognition	Capacity to recognize the information stored from a visual or verbal stimulus	Rey Auditory Verbal Learning - RAVLT
Executive Function	Ability enabling the individual to create plans, goal-setting, strategies, make decisions, monitoring, solving problems, mental flexibility and response initiation and inhibition	Wisconsin Card Sorting Test - WCST
Ideomotor Praxis	Capacity to assemble, build and draw from a mental model	Clock Drawing Test
Constructive Praxis	Capacity to assemble, build and draw from a concrete model	Cubes (WAIS- III)
Naming Ability	Capacity to name objects or pictures	Boston Naming Test - BNT
Verbal Fluency	Capacity to produce a quantity of words, usually within a restricted category	FAS

Data were analyzed using the Statistical Package for the Social Sciences 17.0. All variables were distributed in relation to presence or absence of MetS, using Student's t-test or the Mann-Whitney test.

## RESULTS

Forty-five patients (91.8%) were female, with a mean age of 73.9±5.9 years, and 3.0±1.0 years of schooling. A significant difference (p<0.01) was identified between case and control groups regarding waist circumference measures, fasting plasma glucose measures, HDL-cholesterol and triglycerides levels (Table 1).

**Table 1:** Distribution of means and standard deviation of metabolic parameters in subjects with and without metabolic syndrome.

Parameters	ControlMean±SD	Metabolic SyndromeMean±SD	P
Systolic arterial pressure (mmHg)^a^	133.6±14.8	139.0±22.8	0.33
Diastolic arterial pressure (mmHg)^a^	78.2±10.1	76.4±10.8	0.54
Waist circumference (cm)^b^	91.1±11.8	105.7±13.4	0.000
Fasting plasma glucose (mg/dL)^b^	84.4±9.3	114.7±23.6	0.000
HDL- cholesterol (mg/dL)^a^	66.1±13.7	51.2±13.8	0.000
Triglycerides (mg/dL)^a^	90.0±35.3	140.7±78.4	0.006

a Student's t-test;

b Mann-Whitney U test.

Table 2 describes the cognitive performance of the 49 subjects. All mean scores of the abilities of sustained and alternating attention, executive function and memory (recognition) were impaired compared with standard healthy elderly in both groups of this study, given all had a Z-score lower than -0.7. These data mean that participants' performances were lower than expected for their age, gender and schooling. There was no statistical difference between groups (MetS vs. control).

**Table 2:** Distribution of means and standard deviation of cognitive abilities in subjects with and without metabolic syndrome.

Parameters	Control Mean±SD	Metabolic Syndrome Mean±SD	P
Sustained attention (Z-score)^b^	-3.1±3.5	-4.9±4.4	0.07
Alternating attention (Z- score)^b^	-4.8±5.8	-9.0±19.8	0.15
Working memory (Z-score)^a^	0.5±1.3	0.9±1.3	0.29
Immediate memory (Z-score)^a^	1.1±1.4	1.1±1.0	0.93
Mental flexibility (Z-score)^a^	-0.1±0.8	-0.5±1.1	0.27
Long memory - immediate recall (Z-score)^a^	-0.3±1.4	-0.5±1.1	0.58
Long memory - delayed recall (Z-score)^a^	-0.1±1.1	-0.5±1.2	0.23
Long memory - recognition (Z-score)^a^	-0.7±1.3	-1,1±1.8	0.31
Executive function (Z-score)^b^	-2.1±4.2	-2.9±4.6	0.38
Constructive praxis (weighted)^a^	9.8±2.4	9.7±2.4	0.87
Naming ability (Z-score)^b^	-0.0±1.2	-0.4±1.1	0.15
Verbal fluency (Z-score)^a^	-0.1±0.7	-0.4±0.6	0.09

a Student's t-test;

b Mann-Whitney U test.

## DISCUSSION

MetS affects both sexes and all ages and, in Latin America, the condition increases with age and is always more prevalent in women, especially those from lower social strata.^6,13^ Considering this profile, the study sample was representative of MetS patient characteristics. Another relevant aspect is that the patients were also a representative part of the Brazilian elderly population regarding schooling.^1^ As previously recognized in other Brazilian studies, schooling is an important issue in this field of research because high educational level has a protective affect against cognitive impairment.^14-16^ The lack of Brazilian validated versions of some neuropsychological instruments makes it difficult to elucidate the poor performance of our subjects. Most of the instrument scores (except Wechsler's subtests and Wisconsin Card Sorting Test) were previously obtained from American individuals and the low performance of both groups on these tests could be related to the fact that educational levels of Brazilians are lower than Americans. Notwithstanding, no difference was observed between the groups in the sample for the cognitive performance parameter.

This could indicate that MetS elderly outpatients are more likely to be cognitively preserved, as proposed by previous studies.^10,17^

From a clinical point of view, both case and control groups of elderly subjects were cognitively preserved since they were independent and all came unaccom- panied to the hospital. Therefore, these results are in agreement with previously reported data on preserved and well-controlled MetS aging.^10^ The controversy over the association between cognitive im- pairment and MetS remains unclear in elderly subjects with low schooling. Further studies, particularly those with a prospective design, are necessary to explore cognitive abilities in patients with MetS.
